# The genome sequence of the false flower beetle, *Anaspis frontalis *(Linnaeus, 1758)

**DOI:** 10.12688/wellcomeopenres.23726.1

**Published:** 2025-02-19

**Authors:** Maxwell V. L. Barclay, Svetlana Nikolaeva, Dmitry Telnov, Natural History Museum Genome Acquisition Lab, Darwin Tree of Life Barcoding collective, Wellcome Sanger Institute Tree of Life Management, Samples and Laboratory team, Wellcome Sanger Institute Scientific Operations: Sequencing Operations, Wellcome Sanger Institute Tree of Life Core Informatics team, Tree of Life Core Informatics collective, Darwin Tree of Life Consortium

**Affiliations:** 1 Natural History Museum, London, England, UK; 2 Satbayev University, Almaty, Almaty Province, Kazakhstan; 3 Daugavpils University, Daugavpils, Latvia; 4 Institute of Biology, University of Latvia, Rīga, Latvia

**Keywords:** Anaspis frontalis, false flower beetle, genome sequence, chromosomal, Coleoptera

## Abstract

We present a genome assembly from a specimen of *Anaspis frontalis* (the false flower beetle; Arthropoda; Insecta; Coleoptera; Scraptiidae). The assembly contains two haplotypes with total lengths of 808.55 megabases and 802.05 megabases. Most of haplotype 1 (95.81%) is scaffolded into 8 chromosomal pseudomolecules, including the X chromosome, while haplotype 2 is a scaffold-level assembly. The mitochondrial genome has also been assembled and is 16.47 kilobases in length.

## Species taxonomy

Eukaryota; Opisthokonta; Metazoa; Eumetazoa; Bilateria; Protostomia; Ecdysozoa; Panarthropoda; Arthropoda; Mandibulata; Pancrustacea; Hexapoda; Insecta; Dicondylia; Pterygota; Neoptera; Endopterygota; Coleoptera; Polyphaga; Cucujiformia; Tenebrionoidea; Scraptiidae; *Anaspis*; *Anaspis frontalis* (Linnaeus, 1758) (NCBI:txid433021)

## Background

Scraptiidae (false flower beetles) is a small family of Tenebrionoidea. Two subfamilies, Scraptiinae and Anaspidinae, are recognised in Scraptiidae, both of cosmopolitan distribution ( Lawrence & Ślipiński, 2010). While adult representatives of the subfamily Scraptiinae usually dwell in tree foliage and in cracks of tree bark, adults of the subfamily Anaspidinae are usually found on flowering shrubs and other plants ( Duff, 2020; Levey, 2009, and references therein). The English name of the family, false flower beetles, refers to the ecology of Scraptiinae rather than that of Anaspidinae and likely also highlights the external similarity of the adult false flower beetles with other common flower-visiting coleopterans such as tumbling flower beetles (Mordellidae). *A. frontalis* (Linnaeus, 1758) is the type species of the genus *Anaspis* Geoffroy, 1762. The Palaearctic species of the genus *Anaspis* are placed in six subgenera ( Iwan & Kubisz, 2020). There are about 127 *Anaspis* species and subspecies in the Palaearctic Region (not counting one dubious species) of which 79 occur in geographical Europe ( Iwan & Kubisz, 2020) and 11 species in two subgenera represent the British fauna ( Duff, 2018).

*Anaspis frontalis* is placed in the subfamily Anaspidinae tribe Anaspidini ( Iwan & Kubisz, 2020). The species strongly resembles other congeners in external morphology and the fusiform body shape but is different in the shape and structure of the male genital organs and terminalia, the rufous prothoracic legs, the elongate seventh antennomere, the comparatively short antennomeres 9–10, the terminal antennomere subequal to or hardly longer than penultimate antennomere, and the presence of a paired curved prong on the sternite III ( Duff, 2020; Levey, 2009). Adults of *A. frontalis*, particularly the females, are distinctly larger than any other *Anaspis* species occurring in Britain, and have the front of the head below the antennae usually distinctly yellowish (hence the name frontalis). Colour and other morphological features of *Anaspis* species often vary, the larvae are unknown for most of the species, and identification of taxa, especially those from outside central and northern Europe, is often challenging. The genus *Anaspis* in Britain is taxonomically difficult; this is shown by the fact that the 11 currently valid species known from Britain have more than 25 junior synonyms ( Duff, 2018). It is hoped that genomic information may resolve some taxonomic uncertainties among *Anaspis*, as well as presenting a genome for the type species of a large genus.

*Anaspis frontalis* is a trans-Palaearctic species widely distributed from the Pyrenees throughout Europe (including the Mediterranean and the North) towards western Siberia ( GBIF Secretariat, 2024; Iwan & Kubisz, 2020). Iwan and Kubisz (2020) list the species from 37 countries, 31 European (Austria, Belarus, Belgium, Bosnia and Herzegovina, Bulgaria, Czechia, Denmark, Estonia, Finland, France, Germany, Greece, Hungary, Ireland, Italy, Latvia, Lithuania, Luxemburg, the Netherlands, North Macedonia, Norway, Poland, Romania, most of the European Russia, Slovakia, Slovenia, Spain, Sweden, Switzerland, Ukraine including Crimea ( Odnosum, 1990), and the United Kingdom) but omit Moldova ( Bacal *et al.*, 2013). In Asia, the species is known from most of the boreal zone and is recorded from western and eastern Siberia eastwards to Primorsky Krai of Russia ( Odnosum, 2009), Mongolia, Japan, and Republic of Korea ( Iwan & Kubisz, 2020), as well as the Levant and Turkey ( Iwan & Kubisz, 2020). The species appears less common in hot arid areas, e.g., the Mediterranean, and is mainly absent from the steppe zone of Eurasia, but is common in forested areas including the north of the supercontinent.

*Anaspis frontalis* is a species associated with forests. Larvae of *A. frontalis* are saproxylic and develop in decaying wood of various deciduous trees and are polyphagous, with adults reared from decaying wood of *Acer platanoides*, *Quercus* spp. ( Palm, 1959), and also *Alnus glutinosa*, *Ulmus* spp. (D. Telnov, personal observations in Latvia). The species is described as ubiquist, arboricol, floricol, herbicol ( Koch, 1989) and is bound to decaying wood ( Levey, 2009; Palm, 1959). Adult beetles are anthophilous and occur on the blossoms of various umbellifers, blossoming trees and bushes ( *Rubus* spp., *Sorbus* spp.), in Britain especially on *Crataegus* species ( Duff, 2020). In Britain, adults are reported from April to September ( Duff, 2020) and the sequenced specimen was sampled in the first half of June. In northeastern parts of the range, adults are active from May to September ( Odnosum, 2009).

*Anaspis frontalis* is a widespread and common species in the United Kingdom and recorded in England, Wales and Scotland up to the far North as well as in Ireland ( Duff, 2020; Levey, 2009). The species was not listed in the national Red Data Book ( Shirt, 1987). The present status of the species in the UK is Least Concern (LC) ( Alexander *et al.*, 2014).

The specimen used for sequencing was collected on Bookham Common Surrey, England on 08.vi.2021 by M.V.L. Barclay and S.V. Nikolaeva, from blossom of Dog Rose *Rosa canina* (Rosaceae), and identified by M.V.L. Barclay.

## Genome sequence report

### Sequencing data

**Figure 1.**
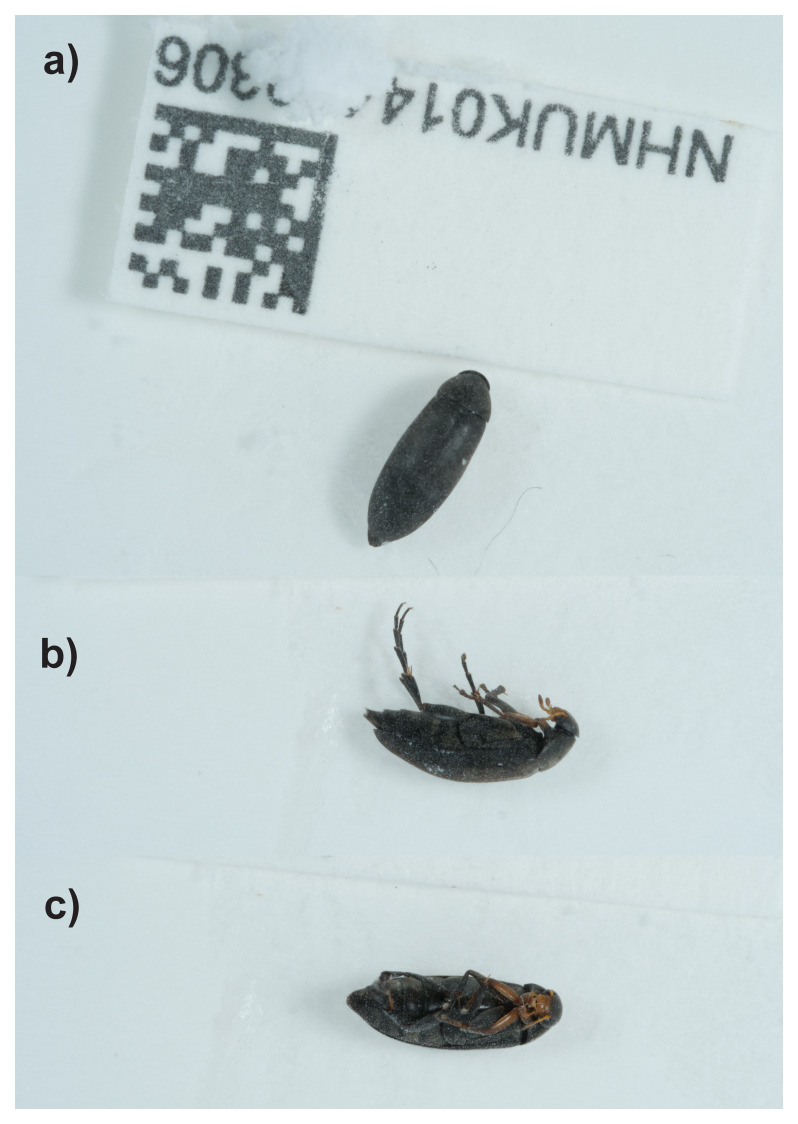
Photograph of the *Anaspis frontalis* (icAnaFron3) specimen used for genome sequencing.

The genome of a specimen of *Anaspis frontalis* ( Figure 1) was sequenced using Pacific Biosciences single-molecule HiFi long reads, generating 21.23 Gb from 2.18 million reads. GenomeScope analysis of the PacBio HiFi data estimated the haploid genome size at 747.39 Mb, with a heterozygosity of 1.47% and repeat content of 44.82%. These values provide an initial assessment of genome complexity and the challenges anticipated during assembly. Based on this estimated genome size, the sequencing data provided approximately 26.0x coverage of the genome. Chromosome conformation Hi-C data produced 230.33 Gb from 1,525.37 million reads. Table 1 summarises the specimen and sequencing information, including the BioProject, study name, BioSample numbers, and sequencing data for each technology.

**Table 1.** Specimen and sequencing data for *Anaspis frontalis*.

Project information			
**Study title**	Anaspis frontalis		
**Umbrella BioProject**	PRJEB73655		
**Species**	*Anaspis frontalis*		
**BioSpecimen**	SAMEA110021804		
**NCBI taxonomy ID**	433021		
Specimen information			
**Technology**	**ToLID**	**BioSample accession**	**Organism part**
**PacBio long read sequencing**	icAnaFron3	SAMEA14448800	head and thorax
**Hi-C sequencing**	icAnaFron3	SAMEA14448801	abdomen
Sequencing information			
**Platform**	**Run accession**	**Read count**	**Base count (Gb)**
**Hi-C Illumina NovaSeq 6000**	ERR12737275	7.28e+08	109.99
**Hi-C Illumina NovaSeq X**	ERR12743662	7.97e+08	120.34
**PacBio Sequel IIe**	ERR12736880	2.18e+06	21.23

### Assembly statistics

The genome was assembled into two haplotypes using Hi-C phasing. Haplotype 1 was curated to chromosome level, while haplotype 2 was assembled to scaffold level and has also been deposited. The assembly was improved by manual curation, which corrected 441 misjoins or missing joins. These interventions reduced the total assembly length by 0.83%, decreased the scaffold count by 7.44%, and increased the scaffold N50 by 1.31%. The final assembly has a total length of 808.55 Mb in 858 scaffolds, with 1,736 gaps, and a scaffold N50 of 99.75 Mb ( Table 2).

**Table 2.** Genome assembly data for *Anaspis frontalis*.

Genome assembly	Haplotype 1	Haplotype 2
Assembly name	icAnaFron3.hap1.1	icAnaFron3.hap2.1
Assembly accession	GCA_964243895.1	GCA_964243875.1
Assembly level	chromosome	scaffold
Span (Mb)	808.55	802.05
Number of contigs	2,594	2,161
Number of scaffolds	858	453
Longest scaffold (Mb)	151.06	None
Assembly metrics * (benchmark)	Haplotype 1	Haplotype 2
Contig N50 length (≥ 1 Mb)	0.66 Mb	0.67 Mb
Scaffold N50 length (= chromosome N50)	99.75 Mb	100.46 Mb
Consensus quality (QV) (≥ 40)	59.2	59.5
*k*-mer completeness	75.44%	75.44%
*Combined k-mer completeness* ( *≥ 95%)*	98.84%	
BUSCO** (S > 90%; D < 5%)	C:98.3%[S:93.7%,D:4.5%], F:0.3%,M:1.5%,n:2,124	C:98.5%[S:93.1%,D:5.4%], F:0.3%,M:1.2%,n:2,124
Percentage of assembly mapped to chromosomes (≥ 90%)	95.81%	-
Sex chromosomes (localised homologous pairs)	X chromosome	-
Organelles (one complete allele)	Mitochondrial genome: 16.47 kb	

* BUSCO scores based on the endopterygota_odb10 BUSCO set using version 5.4.3. C = complete [S = single copy, D = duplicated], F = fragmented, M = missing, n = number of orthologues in comparison.

**Figure 2.**
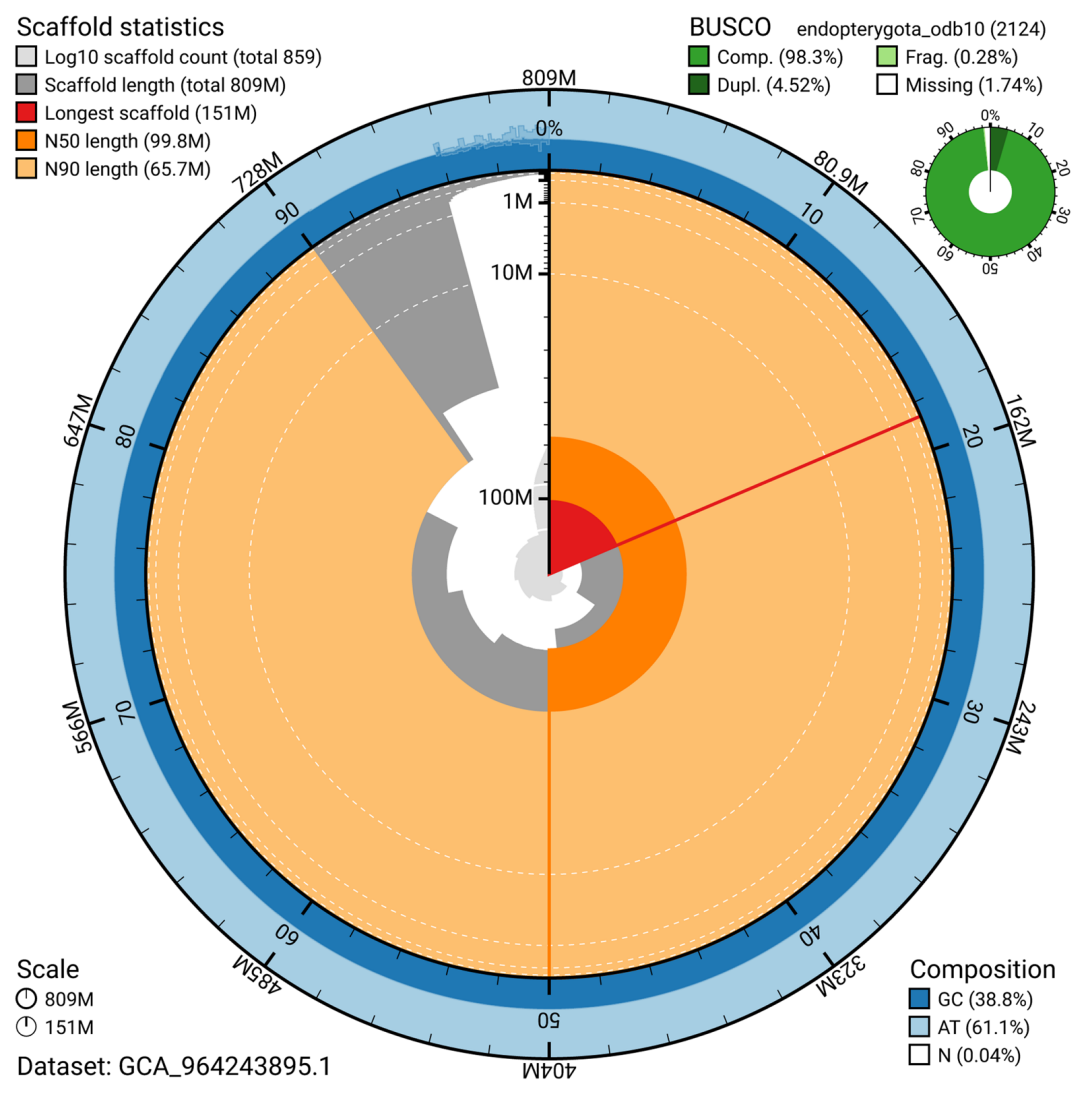
Genome assembly of *Anaspis frontalis*, icAnaFron3.hap1.1: metrics. The BlobToolKit snail plot provides an overview of assembly metrics and BUSCO gene completeness. The circumference represents the length of the whole genome sequence, and the main plot is divided into 1,000 bins around the circumference. The outermost blue tracks display the distribution of GC, AT, and N percentages across the bins. Scaffolds are arranged clockwise from longest to shortest and are depicted in dark grey. The longest scaffold is indicated by the red arc, and the deeper orange and pale orange arcs represent the N50 and N90 lengths. A light grey spiral at the centre shows the cumulative scaffold count on a logarithmic scale. A summary of complete, fragmented, duplicated, and missing BUSCO genes in the set is presented at the top right. An interactive version of this figure is available at https://blobtoolkit.genomehubs.org/view/GCA_964243895.1/dataset/GCA_964243895.1/snail.

**Figure 3.**
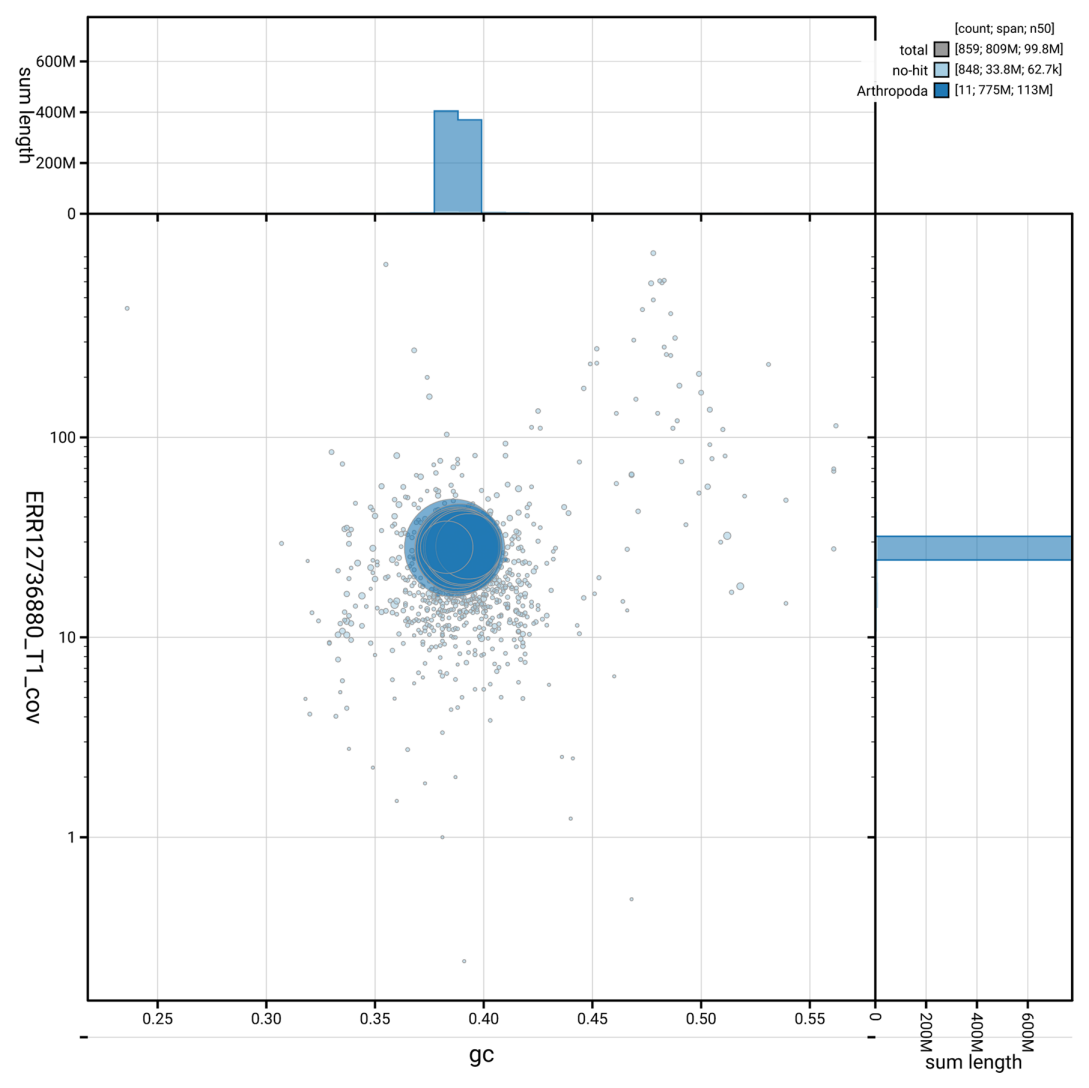
Genome assembly of *Anaspis frontalis*, icAnaFron3.hap1.1: BlobToolKit GC-coverage plot. Blob plot showing sequence coverage (vertical axis) and GC content (horizontal axis). The circles represent scaffolds, with the size proportional to scaffold length and the colour representing phylum membership. The histograms along the axes display the total length of sequences distributed across different levels of coverage and GC content. An interactive version of this figure is available at https://blobtoolkit.genomehubs.org/view/GCA_964243895.1/blob.

**Figure 4.**
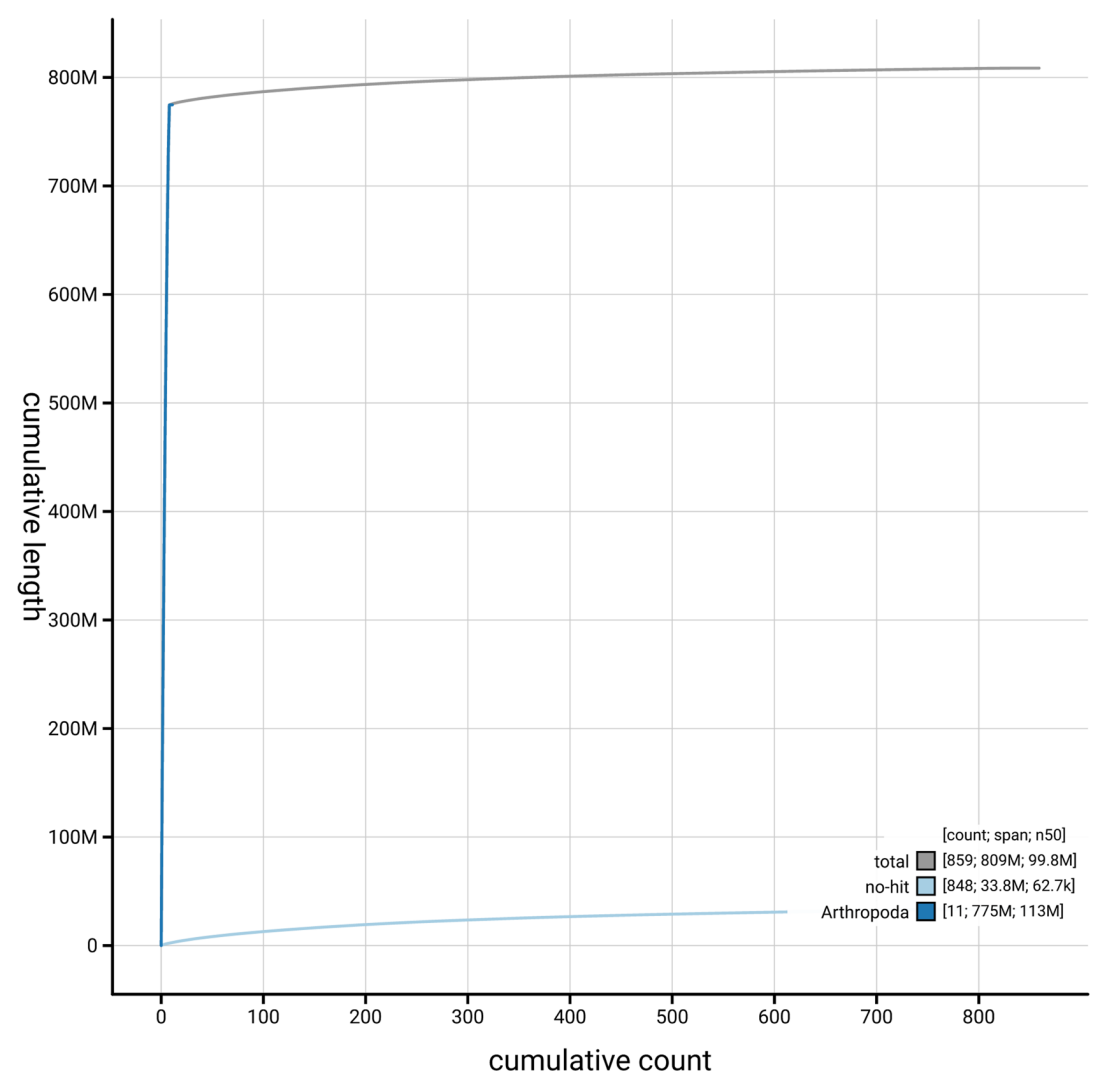
Genome assembly of *Anaspis frontalis* icAnaFron3.hap1.1: BlobToolKit cumulative sequence plot. The grey line shows cumulative length for all scaffolds. Coloured lines show cumulative lengths of scaffolds assigned to each phylum using the buscogenes taxrule. An interactive version of this figure is available at https://blobtoolkit.genomehubs.org/view/GCA_964243895.1/dataset/GCA_964243895.1/cumulative.

The snail plot in Figure 2 provides a summary of the assembly statistics, indicating the distribution of scaffold lengths and other assembly metrics. Figure 3 shows the distribution of scaffolds by GC proportion and coverage. Figure 4 presents a cumulative assembly plot, with separate curves representing different scaffold subsets assigned to various phyla, illustrating the completeness of the assembly.

**Figure 5.**
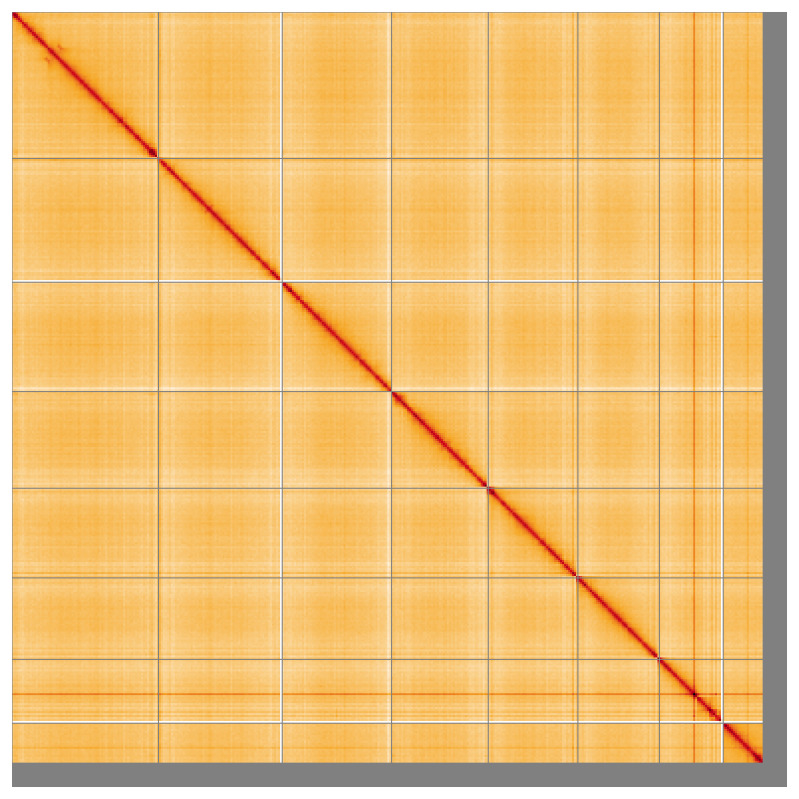
Genome assembly of *Anaspis frontalis* icAnaFron3.hap1.1: Hi-C contact map of the icAnaFron3.hap1.1 assembly, visualised using HiGlass. Chromosomes are shown in order of size from left to right and top to bottom. An interactive version of this figure may be viewed at https://genome-note-higlass.tol.sanger.ac.uk/l/?d=Uf0IdYoBRn-K-sb_tIkbaQ.

Most of the assembly sequence (95.81%) was assigned to 8 chromosomal-level scaffolds. These chromosome-level scaffolds, confirmed by Hi-C data, are named according to size ( Figure 5; Table 3). During curation, chromosome X was assigned by synteny to the genome of *Anaspis regimbarti* (GCA_964204715.1).

**Table 3.** Chromosomal pseudomolecules in the genome assembly of *Anaspis frontalis*, icAnaFron3.

INSDC accession	Name	Length (Mb)	GC%
OZ175968.1	1	151.06	38.5
OZ175969.1	2	127.6	39
OZ175970.1	3	112.8	39
OZ175971.1	4	99.75	39
OZ175972.1	5	92.33	39
OZ175973.1	6	84.09	39
OZ175974.1	7	65.75	39.5
OZ175975.1	X	41.26	38.5
OZ175976.1	MT	0.02	24

The mitochondrial genome was also assembled. This sequence is included as a contig in the multifasta file of the genome submission and as a standalone record in GenBank.

### Assembly quality metrics

The estimated Quality Value (QV) and *k*-mer completeness metrics, along with BUSCO completeness scores, were calculated for each haplotype and the combined assembly. The QV reflects the base-level accuracy of the assembly, while *k*-mer completeness indicates the proportion of expected *k*-mers identified in the assembly. BUSCO scores provide a measure of completeness based on benchmarking universal single-copy orthologues.

For haplotype 1, the estimated QV is 59.2, and for haplotype 2, the QV is 59.5. When the two haplotypes are combined, the assembly achieves an estimated QV of 59.3. The *k*-mer completeness for haplotype 1 is 75.44%, and for haplotype 2 it is 75.44%. When the two haplotypes are combined, the assembly achieves a *k*-mer completeness of 98.84%. BUSCO 5.5.0 analysis using the endopterygota_odb10 reference set ( *n* = 2,124) achieved a completeness score of 98.3% (single = 93.7%, duplicated = 4.5%) for haplotype 1.

Table 2 provides assembly metric benchmarks adapted from Rhie *et al.* (2021) and the Earth BioGenome Project Report on Assembly Standards September 2024. The achieves the EBP reference standard of 5.C.Q59.

## Methods

### Sample acquisition and DNA barcoding

A specimen of *Anaspis frontalis* (specimen ID NHMUK014400306, ToLID icAnaFron3) was collected from Great Bookham Common, England, UK (latitude 51.29, longitude –0.39) on 2021-06-08 by handpicking. The specimen was collected and identified by Maxwell Barclay and Svetlana Nikolaeva (Natural History Museum) and preserved by dry freezing (–80 °C).

The initial identification was verified by an additional DNA barcoding process according to the framework developed by Twyford *et al*. (2024). A small sample was dissected from the specimen and stored in ethanol, while the remaining parts were shipped on dry ice to the Wellcome Sanger Institute (WSI). The tissue was lysed, the COI marker region was amplified by PCR, and amplicons were sequenced and compared to the BOLD database, confirming the species identification ( Crowley *et al.*, 2023). Following whole genome sequence generation, the relevant DNA barcode region was also used alongside the initial barcoding data for sample tracking at the WSI ( Twyford *et al.*, 2024). The standard operating procedures for Darwin Tree of Life barcoding have been deposited on protocols.io ( Beasley *et al.*, 2023).

Metadata collection for samples adhered to the Darwin Tree of Life project standards described by Lawniczak *et al*. (2022).

### Nucleic acid extraction

The workflow for high molecular weight (HMW) DNA extraction at the Wellcome Sanger Institute (WSI) Tree of Life Core Laboratory includes a sequence of procedures: sample preparation and homogenisation, DNA extraction, fragmentation and purification. Detailed protocols are available on protocols.io ( Denton *et al.*, 2023b). The icAnaFron3 sample was prepared for DNA extraction by weighing and dissecting it on dry ice ( Jay *et al.*, 2023). Tissue from the head and thorax was homogenised using a PowerMasher II tissue disruptor ( Denton *et al.*, 2023a). HMW DNA was extracted using the Automated MagAttract v2 protocol ( Oatley *et al.*, 2023a). For ULI PacBio sequencing, DNA was fragmented using the Covaris g-TUBE method ( Oatley *et al.*, 2023c). Sheared DNA was purified by solid-phase reversible immobilisation, using AMPure PB beads to eliminate shorter fragments and concentrate the DNA ( Oatley *et al.*, 2023b). The concentration of the sheared and purified DNA was assessed using a Nanodrop spectrophotometer and Qubit Fluorometer using the Qubit dsDNA High Sensitivity Assay kit. Fragment size distribution was evaluated by running the sample on the FemtoPulse system.

### Hi-C sample preparation

Tissue from the abdomen of the icAnaFron3 sample was processed for Hi-C sequencing at the WSI Scientific Operations core, using the Arima-HiC v2 kit. In brief, 20–50 mg of frozen tissue (stored at –80 °C) was fixed, and the DNA crosslinked using a TC buffer with 22% formaldehyde concentration. After crosslinking, the tissue was homogenised using the Diagnocine Power Masher-II and BioMasher-II tubes and pestles. Following the Arima-HiC v2 kit manufacturer's instructions, crosslinked DNA was digested using a restriction enzyme master mix. The 5’-overhangs were filled in and labelled with biotinylated nucleotides and proximally ligated. An overnight incubation was carried out for enzymes to digest remaining proteins and for crosslinks to reverse. A clean up was performed with SPRIselect beads prior to library preparation. Additionally, the biotinylation percentage was estimated using the Qubit Fluorometer v4.0 (Thermo Fisher Scientific) and Qubit HS Assay Kit and Arima-HiC v2 QC beads.

### Library preparation and sequencing

Library preparation and sequencing were performed at the WSI Scientific Operations core.

***PacBio HiFi***

The sample requires Covaris g-TUBE shearing to approximately 10 kb prior to library preparation. Ultra-low input libraries were prepared using PacBio SMRTbell® Express Template Prep Kit 2.0 and PacBio SMRTbell® gDNA Sample Amplification Kit. To begin, samples were normalised to 20 ng of DNA. Initial removal of single-strand overhangs, DNA damage repair, and end repair/A-tailing were performed per manufacturer’s instructions. From the SMRTbell® gDNA Sample Amplification Kit, amplification adapters were then ligated. A 0.85X pre-PCR clean-up was performed with Promega ProNex beads and the sample was then divided into two for a dual PCR. PCR reactions A and B each followed the PCR programs as described in the manufacturer’s protocol. A 0.85X post-PCR clean-up was performed with ProNex beads for PCR reactions A and B and DNA concentration was quantified using the Qubit Fluorometer v4.0 (Thermo Fisher Scientific) and Qubit HS Assay Kit and fragment size analysis was carried out using the Agilent Femto Pulse Automated Pulsed Field CE Instrument (Agilent Technologies) and gDNA 55kb BAC analysis kit. PCR reactions A and B were then pooled, ensuring the total mass was ≥500 ng in 47.4 μl. The pooled sample then repeated the process for DNA damage repair, end repair/A-tailing and additional hairpin adapter ligation. A 1X clean-up was performed with ProNex beads and DNA concentration was quantified using the Qubit and fragment size analysis was carried out using the Agilent Femto Pulse Automated Pulsed Field CE Instrument (Agilent Technologies). Size selection was performed using Sage Sciences' PippinHT system with target fragment size determined by analysis from the Femto Pulse, usually a value between 4000 and 9000 bp. Size selected libraries were then cleaned-up using 1.0X ProNex beads and normalised to 2 nM before proceeding to sequencing.

Samples were sequenced using the Sequel IIe system (Pacific Biosciences, California, USA). The concentration of the library loaded onto the Sequel IIe was in the range 40–135 pM. The SMRT link software, a PacBio web-based end-to-end workflow manager, was used to set-up and monitor the run, as well as perform primary and secondary analysis of the data upon completion.

***Hi-C***

For Hi-C library preparation, DNA was fragmented using the Covaris E220 sonicator (Covaris) and size selected using SPRISelect beads to 400 to 600 bp. The DNA was then enriched using the Arima-HiC v2 kit Enrichment beads. Using the NEBNext Ultra II DNA Library Prep Kit (New England Biolabs) for end repair, a-tailing, and adapter ligation. This uses a custom protocol which resembles the standard NEBNext Ultra II DNA Library Prep protocol but where library preparation occurs while DNA is bound to the Enrichment beads. For library amplification, 10 to 16 PCR cycles were required, determined by the sample biotinylation percentage. The Hi-C sequencing was performed using paired-end sequencing with a read length of 150 bp on an Illumina NovaSeq X instrument.

### Genome assembly, curation and evaluation

***Assembly***

Prior to assembly of the PacBio HiFi reads, a database of *k*-mer counts ( *k* = 31) was generated from the filtered reads using FastK. GenomeScope2 ( Ranallo-Benavidez *et al.*, 2020) was used to analyse the *k*-mer frequency distributions, providing estimates of genome size, heterozygosity, and repeat content.

The HiFi reads were assembled using Hifiasm in Hi-C phasing mode ( Cheng *et al.*, 2021; Cheng *et al.*, 2022), resulting in a pair of haplotype-resolved assemblies. The Hi-C reads were mapped to the primary contigs using bwa-mem2 ( Vasimuddin *et al.*, 2019). The contigs were further scaffolded using the provided Hi-C data ( Rao *et al.*, 2014) in YaHS ( Zhou *et al.*, 2023) using the --break option for handling potential misassemblies. The scaffolded assemblies were evaluated using Gfastats ( Formenti *et al.*, 2022), BUSCO ( Manni *et al.*, 2021) and MERQURY.FK ( Rhie *et al.*, 2020).

The mitochondrial genome was assembled using MitoHiFi ( Uliano-Silva *et al.*, 2023), which runs MitoFinder ( Allio *et al.*, 2020) and uses these annotations to select the final mitochondrial contig and to ensure the general quality of the sequence.

***Assembly curation***

The assembly was decontaminated using the Assembly Screen for Cobionts and Contaminants (ASCC) pipeline (article in preparation). Flat files and maps used in curation were generated in TreeVal ( Pointon *et al.*, 2023). Manual curation was primarily conducted using PretextView ( Harry, 2022), with additional insights provided by JBrowse2 ( Diesh *et al.*, 2023) and HiGlass ( Kerpedjiev *et al.*, 2018). Scaffolds were visually inspected and corrected as described by Howe *et al*. (2021). Any identified contamination, missed joins, and mis-joins were corrected, and duplicate sequences were tagged and removed. The X chromosome was assigned by synteny analysis. The curation process is documented at https://gitlab.com/wtsi-grit/rapid-curation (article in preparation).

***Assembly quality assessment***

The Merqury.FK tool ( Rhie *et al.*, 2020), run in a Singularity container ( Kurtzer *et al.*, 2017), was used to evaluate *k*-mer completeness and assembly quality for the primary and alternate haplotypes using the *k*-mer databases ( *k* = 31) that were computed prior to genome assembly. The analysis outputs included assembly QV scores and completeness statistics.

A Hi-C contact map was produced for the final version of the assembly. The Hi-C reads were aligned using bwa-mem2 ( Vasimuddin *et al.*, 2019) and the alignment files were combined using SAMtools ( Danecek *et al.*, 2021). The Hi-C alignments were converted into a contact map using BEDTools ( Quinlan & Hall, 2010) and the Cooler tool suite ( Abdennur & Mirny, 2020). The contact map is visualised in HiGlass ( Kerpedjiev *et al.*, 2018).

The blobtoolkit pipeline is a Nextflow port of the previous Snakemake Blobtoolkit pipeline ( Challis *et al.*, 2020). It aligns the PacBio reads in SAMtools and minimap2 ( Li, 2018) and generates coverage tracks for regions of fixed size. In parallel, it queries the GoaT database ( Challis *et al.*, 2023) to identify all matching BUSCO lineages to run BUSCO ( Manni *et al.*, 2021). For the three domain-level BUSCO lineages, the pipeline aligns the BUSCO genes to the UniProt Reference Proteomes database ( Bateman *et al.*, 2023) with DIAMOND blastp ( Buchfink *et al.*, 2021). The genome is also divided into chunks according to the density of the BUSCO genes from the closest taxonomic lineage, and each chunk is aligned to the UniProt Reference Proteomes database using DIAMOND blastx. Genome sequences without a hit are chunked using seqtk and aligned to the NT database with blastn ( Altschul *et al.*, 1990). The blobtools suite combines all these outputs into a blobdir for visualisation.

The blobtoolkit pipeline was developed using nf-core tooling ( Ewels *et al.*, 2020) and MultiQC ( Ewels *et al.*, 2016), relying on the Conda package manager, the Bioconda initiative ( Grüning *et al.*, 2018), the Biocontainers infrastructure ( da Veiga Leprevost *et al.*, 2017), as well as the Docker ( Merkel, 2014) and Singularity ( Kurtzer *et al.*, 2017) containerisation solutions.

Table 4 contains a list of relevant software tool versions and sources.

**Table 4.** Software tools: versions and sources.

Software tool	Version	Source
BEDTools	2.30.0	https://github.com/arq5x/bedtools2
BLAST	2.14.0	ftp://ftp.ncbi.nlm.nih.gov/blast/executables/blast+/
BlobToolKit	4.3.9	https://github.com/blobtoolkit/blobtoolkit
BUSCO	5.5.0	https://gitlab.com/ezlab/busco
bwa-mem2	2.2.1	https://github.com/bwa-mem2/bwa-mem2
Cooler	0.8.11	https://github.com/open2c/cooler
DIAMOND	2.1.8	https://github.com/bbuchfink/diamond
fasta_windows	0.2.4	https://github.com/tolkit/fasta_windows
FastK	427104ea91c78c3b8b8b49f1a7d6bbeaa869ba1c	https://github.com/thegenemyers/FASTK
Gfastats	1.3.6	https://github.com/vgl-hub/gfastats
GoaT CLI	0.2.5	https://github.com/genomehubs/goat-cli
Hifiasm	0.19.8-r603	https://github.com/chhylp123/hifiasm
HiGlass	44086069ee7d4d3f6f3f0012569789ec138f42b84 aa44357826c0b6753eb28de	https://github.com/higlass/higlass
MerquryFK	d00d98157618f4e8d1a9190026b19b471055b22e	https://github.com/thegenemyers/MERQURY.FK
Minimap2	2.24-r1122	https://github.com/lh3/minimap2
MitoHiFi	3	https://github.com/marcelauliano/MitoHiFi
MultiQC	1.14, 1.17, and 1.18	https://github.com/MultiQC/MultiQC
NCBI Datasets	15.12.0	https://github.com/ncbi/datasets
Nextflow	23.10.0	https://github.com/nextflow-io/nextflow
PretextView	0.2.5	https://github.com/sanger-tol/PretextView
samtools	1.19.2	https://github.com/samtools/samtools
sanger-tol/ascc	-	https://github.com/sanger-tol/ascc
sanger-tol/blobtoolkit	0.5.1	https://github.com/sanger-tol/blobtoolkit
Seqtk	1.3	https://github.com/lh3/seqtk
Singularity	3.9.0	https://github.com/sylabs/singularity
TreeVal	1.2.0	https://github.com/sanger-tol/treeval
YaHS	1.2a.2	https://github.com/c-zhou/yahs

### Wellcome Sanger Institute – Legal and Governance

The materials that have contributed to this genome note have been supplied by a Darwin Tree of Life Partner. The submission of materials by a Darwin Tree of Life Partner is subject to the **‘Darwin Tree of Life Project Sampling Code of Practice’**, which can be found in full on the Darwin Tree of Life website here. By agreeing with and signing up to the Sampling Code of Practice, the Darwin Tree of Life Partner agrees they will meet the legal and ethical requirements and standards set out within this document in respect of all samples acquired for, and supplied to, the Darwin Tree of Life Project.

Further, the Wellcome Sanger Institute employs a process whereby due diligence is carried out proportionate to the nature of the materials themselves, and the circumstances under which they have been/are to be collected and provided for use. The purpose of this is to address and mitigate any potential legal and/or ethical implications of receipt and use of the materials as part of the research project, and to ensure that in doing so we align with best practice wherever possible. The overarching areas of consideration are:

•   Ethical review of provenance and sourcing of the material

•   Legality of collection, transfer and use (national and international)

Each transfer of samples is further undertaken according to a Research Collaboration Agreement or Material Transfer Agreement entered into by the Darwin Tree of Life Partner, Genome Research Limited (operating as the Wellcome Sanger Institute), and in some circumstances other Darwin Tree of Life collaborators.

## Data Availability

European Nucleotide Archive: Anaspis frontalis. Accession number PRJEB73655; https://identifiers.org/ena.embl/PRJEB73655. The genome sequence is released openly for reuse. The *Anaspis frontalis* genome sequencing initiative is part of the Darwin Tree of Life (DToL) project. All raw sequence data and the assembly have been deposited in INSDC databases. The genome will be annotated using available RNA-Seq data and presented through the Ensembl pipeline at the European Bioinformatics Institute. Raw data and assembly accession identifiers are reported in Table 1 and Table 2.

## Author information

Members of the Natural History Museum Genome Acquisition Lab are listed here: https://doi.org/10.5281/zenodo.12159242.

Members of the Darwin Tree of Life Barcoding collective are listed here: https://doi.org/10.5281/zenodo.12158331.

Members of the Wellcome Sanger Institute Tree of Life Management, Samples and Laboratory team are listed here: https://doi.org/10.5281/zenodo.12162482.

Members of Wellcome Sanger Institute Scientific Operations: Sequencing Operations are listed here: https://doi.org/10.5281/zenodo.12165051.

Members of the Wellcome Sanger Institute Tree of Life Core Informatics team are listed here: https://doi.org/10.5281/zenodo.12160324.

Members of the Tree of Life Core Informatics collective are listed here: https://doi.org/10.5281/zenodo.12205391.

Members of the Darwin Tree of Life Consortium are listed here: https://doi.org/10.5281/zenodo.4783558.
