# Supplementary material for: The genome sequence of the false flower beetle, Anaspis frontalis (Linnaeus, 1758)
Source: Wellcome Open Res. 2025 Feb 19;10:82. [Version 1] doi: 10.12688/wellcomeopenres.23726.1 (PMC11909497; doi:10.12688/wellcomeopenres.23726.1)
Supplement: Peer review history

# Reviewer report: Reviewer response for version 1

**DOI:** 10.21956/wellcomeopenres.26174.r119891

**Published:** 2025-03-14

**Authors:** Timothy PL Smith

**Affiliations:** 1 USDA-ARS, U.S. Meat Animal Research Center, Clay Center, USA

The description of the process and results of genome sequencing for an individual false flower beetle are sufficient to understand and potentially replicate the study.  Mildly surprising that the contigs aren't longer given the reasonable GC content (for insects) but this could be impact of the ultra-low input protocol that I don't have direct experience with.  Very minor comments: in the background section there appear to be missing parentheses i.e. (Geoffrey, 1762).  Also, in last paragraph before Methods, "The achieves the EBP reference standard.." should probably say "The **assembly** achieves..."

Are sufficient details of methods and materials provided to allow replication by others?

Yes

Is the rationale for creating the dataset(s) clearly described?

Yes

Are the datasets clearly presented in a useable and accessible format?

Yes

Are the protocols appropriate and is the work technically sound?

Yes

Reviewer Expertise:

genome and metagenome assembly and analysis (not particularly insects)

I confirm that I have read this submission and believe that I have an appropriate level of expertise to confirm that it is of an acceptable scientific standard.

# Reviewer report: Reviewer response for version 1

**DOI:** 10.21956/wellcomeopenres.26174.r119241

**Published:** 2025-02-28

**Authors:** Hume B Douglas

**Affiliations:** 1 Agriculture and Agri-Food Canada, Ottawa, Canada

The work is great. I have a few suggestions for readability.

 Please add beetle family name to title

 Please state in paragraph 1 that Anaspis is the type genus of SF Anaspidinae This won't be obvious to non-taxonomists

 Please add a statement about literature etc. used to identify the specimen following Packer *et al.*(2018)

 Also,  please mention whether any voucher specimen remains and where it will be deposited.

 The statement “The English name of the family, false flower beetles, refers to the ecology of Scraptiinae rather than that of Anaspidinae” is a little confusing since you say that Anaspidines are more likely to be found on flowering shrubs etc.

 The background information about only Palaearctic species might make readers think this is only a Palearctic genus. Please mention other world regions (e.g. Nearctic) also. This is not only a local European-focused publication, right?

 What is the global range of *Anaspis frontalis*? OK, you mention this in paragraph 3, but it would have been useful to at least know it was from the trans Palaearctic earlier. Please mention this early that this is a Palaearctic beetle.

 This will be an essential resource for future scraptiid studies, and maybe for tenebrionoids in general.

 Thanks

Are sufficient details of methods and materials provided to allow replication by others?

Yes

Is the rationale for creating the dataset(s) clearly described?

Partly

Are the datasets clearly presented in a useable and accessible format?

Yes

Are the protocols appropriate and is the work technically sound?

Yes

Reviewer Expertise:

Beetle taxonomy (not particularly genomes)

I confirm that I have read this submission and believe that I have an appropriate level of expertise to confirm that it is of an acceptable scientific standard.

## References

[1] : Validating taxonomic identifications in entomological research. *Insect Conservation and Diversity* .2018;11(1) : 10.1111/icad.12284 1-12 10.1111/icad.12284
